# *Ganoderma* spore powder contains little triterpenoids

**DOI:** 10.1186/s13020-020-00391-1

**Published:** 2020-10-12

**Authors:** Mei-Ting Liu, Ling-Xiao Chen, Jing Zhao, Shao-Ping Li

**Affiliations:** State Key Laboratory of Quality Research in Chinese Medicine, Institute of Chinese Medical Sciences, University of Macau, Macao, China

**Keywords:** *Ganoderma* spore powder, Triterpenoids, Bionic extraction, Artificial gastroenteric fluid, HPTLC, LC–QTOF–MS

## Abstract

**Background:**

*Ganoderma* spore is a minuscule germ cell ejected from *Ganoderma* gills during its growth maturity period, it has been considered with high exploitable potential in health-care products manufacture.

**Methods:**

After testing sporoderm-broken rate, the triterpenoids in 12 batches of broken and unbroken *Ganoderma* spore powder (GSP) samples were compared with *Ganoderma lucidum* fruiting body (GL) by high performance thin-layer chromatography (HPTLC) and further verified by liquid chromatography coupled with electrospray ionization quadrupole-time-of-flight mass spectrometry (LC–QTOF–MS); meanwhile, the dissolution of triterpenoids after bionic extraction was also investigated by HPTLC.

**Results:**

The sporoderm-broken rate of all the broken GSP samples was over 85%. The relative peak area of triterpenoids in GSP samples were lower than 50% of that in fruiting body, and the dissolution of triterpenoids in artificial gastrointestinal fluid was lower than in methanol.

**Conclusions:**

This study demonstrated that there were little triterpenoids in GSP. Triterpenoids in GSP also seldom be dissolved in artificial gastrointestinal fluid.

## Background

*Ganoderma*, well-known as “Lingzhi” in China, is one of the most famous traditional Chinese medicines. Nowadays, only *Ganoderma lucidum* and *Ganoderma sinense* are officially recorded as “Lingzhi” in Chinese Pharmacopoeia. *Ganoderma* has abundant with active components, such as polysaccharides [1], triterpenoids [2], sterols [3], fatty acids [4] and protein [5]. Among them, triterpenoids are the main active ingredients in *Ganoderma* and one of the Chinese Pharmacopoeia markers, which have anti-viral, anti-tumor and anti-inflammatory activities [6, 7].

*Ganoderma* spore is a minuscule germ cell ejected from *Ganoderma* gills during its growth maturity period. There is a long history behind the spore known as the essence of *Ganoderma*, which reserves all the genetic effective ingredients and has lots of pharmacological activities too, such as immunomodulatory effects [8, 9]. However, spore has two very hard chitin spore walls which make the internal activity components difficult to be absorbed and digested in human gastrointestinal tract. In order to improve the utilization of *Ganoderma* spore powder (GSP), it is essential to break spore walls. Several wall-breaking methods are developed continually, such as ultrasonication with low temperature [10], supercritical carbon dioxide breaking [11] and high-speed centrifugal shearing pulverizer [12]. After breaking the walls, the dissolution rate of the internal ingredients can be increased [13], so its adsorption efficiency of human body can be increased too [14]. Therefore, the application of GSP in health-care products is gradually more than that of fruiting body [15]. According to statistics from related report [16], between 2005 and 2015, 40% of *Ganoderma* health-care products were made from GSP and have been promoted with good medicinal effects. For example, broken GSP capsule was a good anti-tumor auxiliary drug [17] and GSP chewable tablet had the effect on enhancing immune function [18]. Nowadays, GSP has good economic benefits and its product industry is flourishing.

However, the studies about triterpenoids in *Ganoderma* spore still have contradiction, several reports showed that the content of triterpenoids in spore were higher than fruiting body while some reports held opposite views [15, 19]. At present, there are two methods to detect triterpenoids in *Ganoderma* spore: HPLC [15] and colorimetric method of using vanillin-perchloric acid [20]. As triterpenoids are main small molecular effective components in *Ganoderma*, it is necessary to clarify the situation of triterpenoids in GSP.

In this work, the triterpenoids in 12 batches of GSP were compared with GL fruiting body by HPTLC after testing sporoderm-broken rate and further verified by LC–QTOF–MS. Besides, the dissolution of triterpenoids by bionic extraction were also investigated by HPTLC.

## Materials and methods

### *Ganoderma* samples and chemicals

All* Ganoderma* samples were listed in Table 1, including one batch of *Ganoderma lucidum* fruiting body (GL) was collected from Zhejiang Province of China, 12 batches of *Ganoderma* spore powder (GSP-01 to GSP-12) were collected from different regions of China. The voucher specimens were stored at the Institute of Chinese Medical Sciences, University of Macau, Macao, China. Methanol, dichloromethane, ethyl acetate, n-hexane and formic acid were highest available purity (Merck, Darmstadt, Germany), deionized water was produced by a Millipore Milli-Q Plus system (Millipore, Bedford, USA).

**Table 1:** Information and sporoderm-broken rate (%) of GSP samples

Codes	Details	Origins	Sporoderm-broken rate
GL	*Ganoderma lucidum* fruiting body	Zhejiang	/
GSP-01	Broken *Ganoderma* spore powder	Taishan, Shandong	95.76
GSP-02	Unbroken *Ganoderma* spore powder	Taishan, Shandong	/
GSP-03	Broken *Ganoderma* spore powder	Dongbei	88.29
GSP-04	Unbroken *Ganoderma* spore powder	Dongbei	/
GSP-05	Broken *Ganoderma* spore powder	Yunnan	89.83
GSP-06	Unbroken *Ganoderma* spore powder	Yunnan	/
GSP-07	Broken *Ganoderma* spore powder	Guanxian, Shandong	85.54
GSP-08	Unbroken *Ganoderma* spore powder	Guanxian, Shandong	/
GSP-09	Broken *Ganoderma* spore powder	Jinzhai, Anhui	85.61
GSP-10	Unbroken *Ganoderma* spore powder	Jinzhai, Anhui	/
GSP-11	Broken *Ganoderma* spore powder	Longquan, Zhejiang	100.00
GSP-12	Unbroken *Ganoderma* spore powder	Longquan, Zhejiang	/

### Sporoderm-broken rate of GSP

Powder of each sample (2.0 mg) was soaked in 1.0 mL of 30% ethanol. Before observing, each mixture was fully oscillated, taken 10 μL to observed under 40-fold objective lens (Olympus IX73 Inverted Microscope System, Tokyo, Japan). Each mixture was observed three times and averaged, samples from a same origin were compared, sporoderm-broken rate of broken GSP samples was calculated by following equation:

$$ Sporoderm{ - }broken \, rate\left( \% \right) = 100 - N_{2} /N_{1} *100 $$

(N_1_ = the average of spore from unbroken GSP sample; N_2_ = the average of unbroken spore from broken GSP sample).

### Extraction of *Ganoderma* samples

Each sample (1.0 g) was soaked in 10.0 mL of methanol for 1 h and extracted by the ultrasonic extractor (Bransonic Branson 8510, Danbury, USA) for 45 min (room temperature). Then the extract was centrifuged at 4500× rcf for 10 min (ThermoFisher Heraeus Multifuge X3R Centrifuge, Osterode am Harz, Germany). Supernatant was evaporated to desiccation under vacuum by rotary evaporator (Büchi Rotavapor R-205, Flawil, Switzerland), and residue was re-dissolved in 1.0 mL of methanol. After centrifugation at 16,000× rcf for 5 min (Eppendorf AG Centrifuge 5415D, Hamburg, Germany) and filtration through a 0.45 μm membrane filter, the supernatant was used for HPTLC analysis subsequently.

### Bionic extraction

Artificial gastrointestinal fluid was prepared by the 2015 Chinese Pharmacopoeia Part IV general principles as followed: Artificial gastric fluid: 16.4 mL of dilute hydrochloric acid was added about 800 mL of water and 10 g of pepsin, and the mixture was diluted with water to 1000 mL. Artificial intestinal fluid: 6.8 g of potassium dihydrogen phosphate was dissolved with 500 mL of water, and the pH was adjusted to 6.8 with 0.1 mol/L sodium hydroxide solution; meanwhile, 10 g of trypsin was dissolved with an appropriate amount of water, then the mixture was diluted with water to 1000 mL.

Each sample (1.0 g) was soaked in 10.0 mL of artificial gastric fluid and incubated by the thermostatic oscillation incubator (Eppendorf AG Thermomixer comfort, Hamburg, Germany) for 3 h (37 ℃, 150× rpm). Then the extract was centrifuged at 4500× rcf for 10 min. The supernatant was evaporated to desiccation under vacuum using rotary evaporator, and the residue was re-dissolved in 1.0 mL of methanol. After centrifugation at 16,000× rcf for 5 min and filtration through a 0.45 μm membrane filter, the supernatant was used for HPTLC analysis subsequently.

Then the residues were immersed in 10.0 mL of artificial intestinal fluid and incubated by the thermostatic oscillation incubator for 6 h (37 ℃, 150× rpm). Then the extract was centrifuged at 4500× rcf for 10 min. The supernatant was evaporated to desiccation under vacuum using rotary evaporator, and the residue was re-dissolved in 1.0 mL of methanol. After centrifugation at 16,000× rcf for 5 min and filtration through a 0.45 μm membrane filter, the supernatant was used for HPTLC analysis subsequently. Finally, the residues were extracted by methanol according to the section of “Extraction of *Ganoderma* samples”.

### HPTLC analysis of triterpenoids

All samples (7 μL) were formed into band shape on a 20 × 10 cm silica-gel 60 plate (Merck, Darmstadt, Germany) with an automatic HPTLC sampler (CAMAG ATS4, Muttenz, Switzerland). Bands were 8 mm wide, 6 mm apart and at 10 mm from the bottom edge. The plate was developed to 90 mm with dichloromethane/ethyl acetate/methanol/formic acid, 30:20:1:1 (*v/v/v/v*) as developing solvent at room temperature and 88% relative humidity, after pre-saturation in developing solvent vapor for 15 min. Then the developed plates were colorized by spraying 10% (*v/v*) sulfuric acid in ethanol and heated at 105 ℃ for 5 min on a plate heater (KEZHE-SP-III, Shanghai, China). The plate was photographed under daylight and UV 365 nm respectively, and the figure results were integrated by grayscale scanning (GelAnalyzer2010a, Debrecen, Hungary).

### LC–QTOF–MS analysis of triterpenoids

LC–QTOF–MS analysis was performed according to our previous study [21]. Briefly, an Ultimate 3000 UHPLC system equipped with Ultimate 3000 degasser, pump, RS autosampler, and RS column compartment, coupled with diode-array detector (Thermo Fisher, Osterode am Harz, Germany). Waters Acquity BEH C_18_ column (2.1 × 150 mm i.d., 1.7 μm, Waters, Milford, MA, USA) was used for sample separation. The mobile phase was consisted of 0.2% acetic acid in water (A) and acetonitrile (B) with gradient elution: 0–8 min, 3% B-22% B; 8–25 min, 22% B-25% B; 25–40 min, 25% B-35% B; 40–65 min, 35% B-100% B, 65–70 min, 100% B. The flow rate was 0.3 mL/min, injection volume was 1 μL, column temperature was 25 ℃ and detection wavelength was set at 257 nm.

A high-resolution impact HD QTOF mass spectrometer (Bruker Daltonik GmbH, Bremen, Germany) equipped with an electrospray ionization (ESI) source was operated in the negative ion mode. The mass range was set at *m/z* 100–1500 in the full scan mode. The capillary voltage was set at 3500 V. The fragmentation mode was CID. The source temperature was set at 250 ℃. Nitrogen was used as the drying gas. The gas flow rate was set at 8 L/min. MS^2^ data analysis of the three highest intensive ion fragments was intelligently performed in real time.

## Results

### Sporoderm-broken rate of GSP

As shown by objective lens, broken spore had brown translucent fragmented contents, and unbroken spore had double intact plump walls. The sporoderm-broken rate of all the broken GSP samples was over 85% (Table 1), thus, it could be considered that all the broken GSP samples were broken completely. Furthermore, filiform mycelia could not be observed in the samples, which meant that there was no fruiting body powder mixed into GSP samples.

### HPTLC analysis of triterpenoids in GSP

**Fig. 1:**
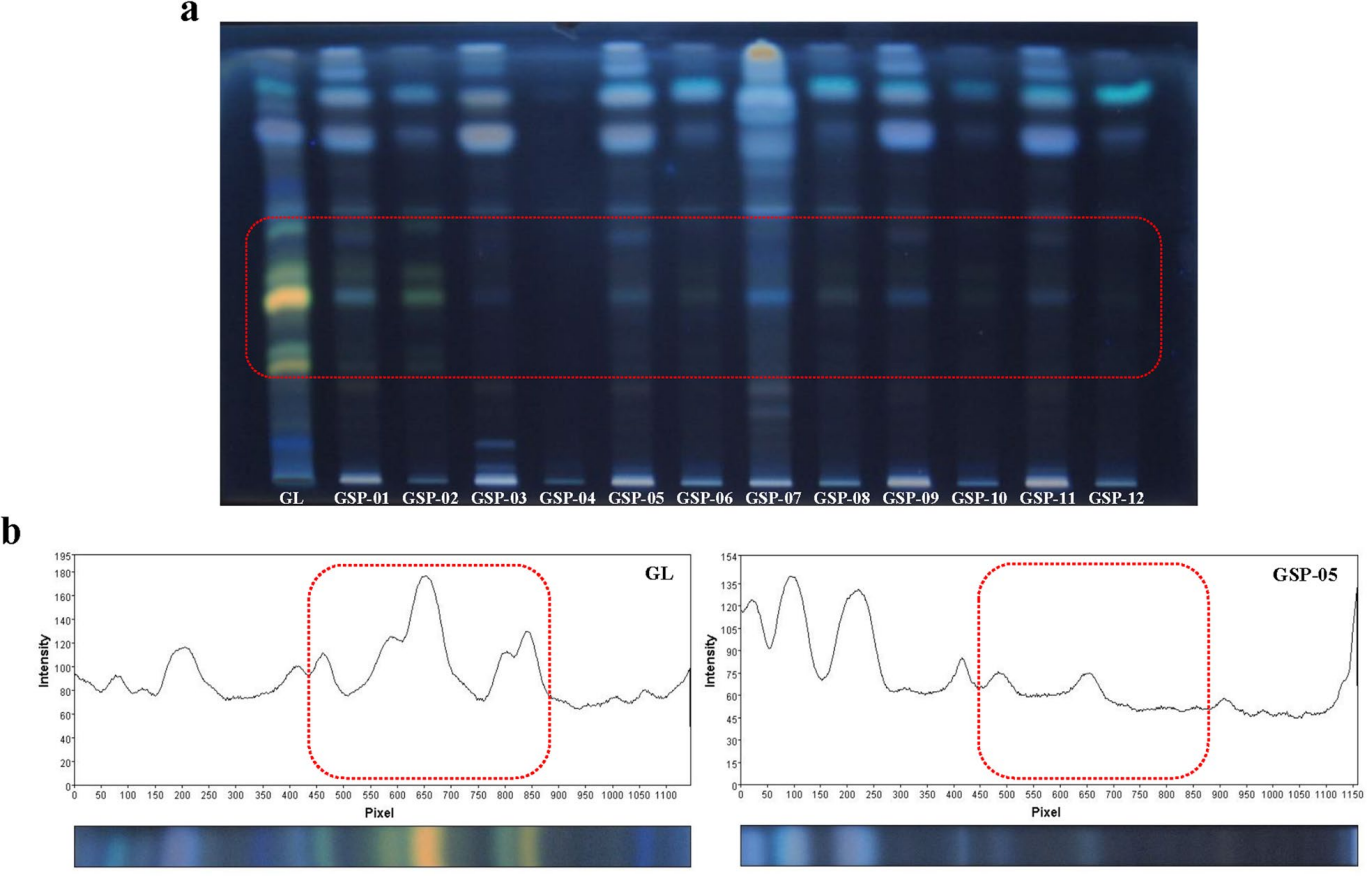
HPTLC fingerprint chromatogram of GSP under UV 365 nm (**a**). Grayscale scanning integral results of GL and GSP-05 (**b**)

HPTLC fingerprints of triterpenoids in 12 batches of GSP at UV 365 nm was shown in Fig. 1a. GL was taken as reference and demonstrated that it supplied yellow or green bands at triterpenoids area (red dotted line part, Rf = 0.27–0.60). However, most of GSP samples just had some trace of bands at this part and the colors were different from those of fruiting body (except GSP-01 and GSP-02), which revealed that these components corresponding to the bands was not as same as the triterpenoids in fruiting body, although their Rf value were similar. As HPTLC fingerprints at UV 365 nm is more sensitive and clearer than under UV 254 nm and daylight [22], it was chosen for image grayscale analysis. Triterpenoids in GSP samples were compared with GL, specifically, by setting the triterpenoids peak area of GL as standard and calculating the relative peak area between GL and each GSP sample. Grayscale scanning integral results of GL and GSP-05 were shown in Fig. 1b. As demonstrated in Table 2, whether the spore walls were broken or not, the triterpenoids peak areas of all GSP samples were lower than 50% of that in fruiting body. Spore wall-breaking process was helpful to dissolve internal ingredients, however, *p* value of triterpenoids peak area was 0.096 (*p* > 0.05), which suggested that there was no significant difference between broken and unbroken GSP samples in triterpenoids. This result might attribute to the GSP samples contained little triterpenes.

**Table 2:** Relative peak area (%) of triterpenoids in GSP samples by HPTLC

Codes	Methanol	Artificial gastric fluid	Artificial intestinal fluid	Residues
GL	100.00	/	/	/
GSP-01	43.50	0.00	27.18	31.95
GSP-02	36.87	0.00	26.59	16.99
GSP-03	7.27	0.00	13.29	18.35
GSP-04	0.00	0.00	0.00	0.00
GSP-05	25.23	0.00	7.20	23.65
GSP-06	26.50	0.00	0.00	10.76
GSP-07	27.92	0.00	14.98	22.94
GSP-08	24.67	0.00	0.00	5.67
GSP-09	15.84	0.00	7.91	14.90
GSP-10	17.20	0.00	0.00	0.00
GSP-11	18.59	0.00	0.00	19.16
GSP-12	14.36	0.00	0.00	0.00

/ meant undetected

### LC–QTOF–MS further verification of triterpenoids

**Fig. 2:**
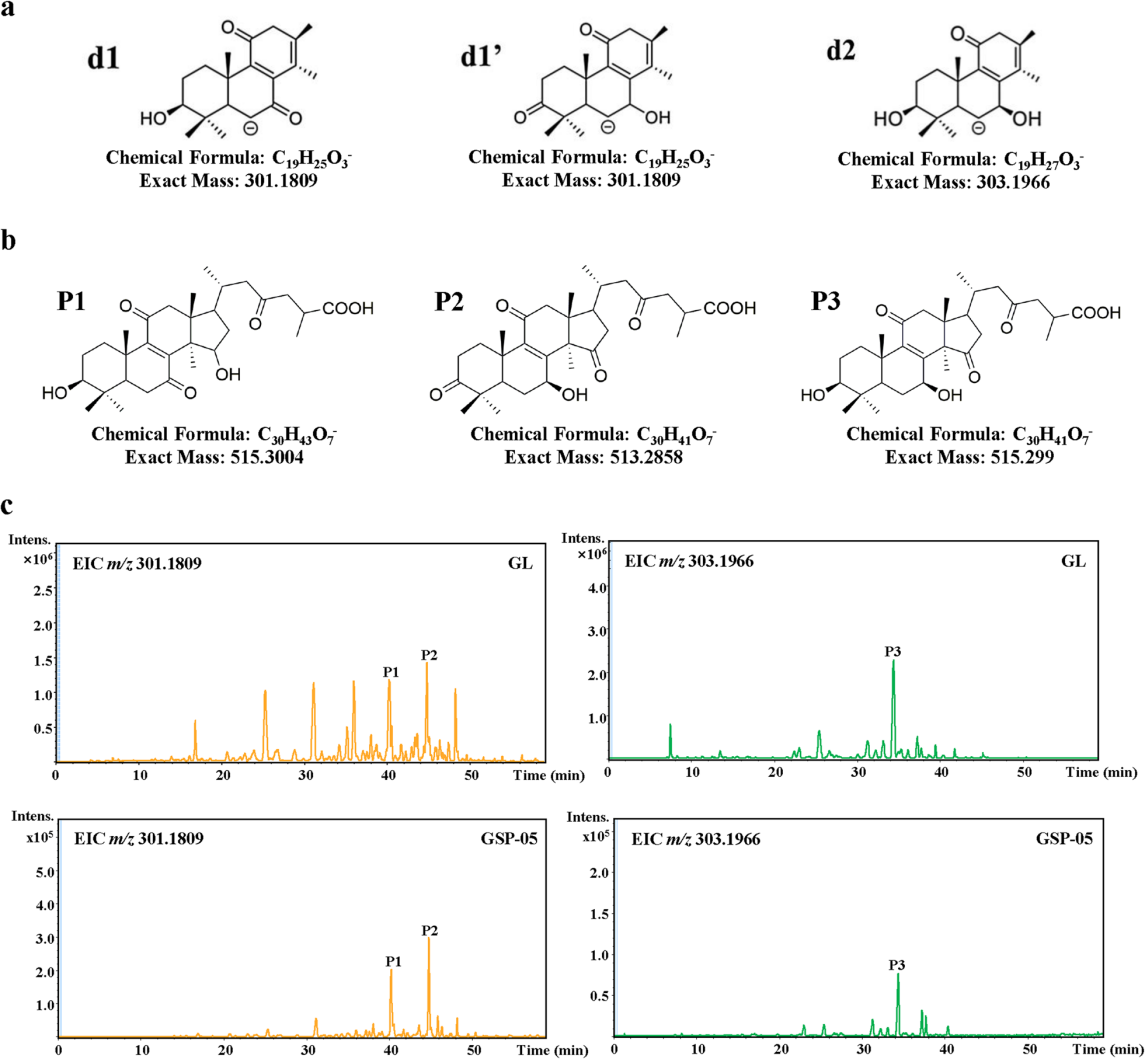
Three major fragment ions of oxygenated tetracyclic triterpenoid (**a**). Possible structures of P1, P2 and P3 (**b**). Extraction ion chromatograms (MS^2^) of GL and GSP-05 (**c**)

As the sensitivity of HPTLC was not high enough, LC–QTOF–MS was used to further verify the results. In previous studies [21, 23], oxygenated tetracyclic triterpenoid was the main type of triterpenoids in *Ganoderma*, and major specific fragment ions for identification of oxygenated tetracyclic triterpenoid were *m/z* 301.1809 (d1 and d1′ type, Fig. 2a) and *m/z* 303.1966 (d2 type, Fig. 2a). P1, P2 and P3 were three main peaks of these type triterpenoid, which detected in GL and representative broken GSP samples, so P1–P3 were representative for profiling the triterpenoids in GSP samples. As listed in Table 3, the main ions in P1 was *m/z* 515.3004 (d1 type), P2 was *m/z* 513.2858 (d1′ type) and the main ions in P3 was *m/z* 515.2990 (d2 type). Furthermore, the proposed fragmentation pathway was referred to our previous study [21], the possible structures of P1, P2 and P3 was shown in Fig. 2b, respectively. The extraction ion chromatograms (MS^2^) of GL and GSP-05 were shown in Fig. 2c, respectively. Relative total peak areas of d1, d1′ and d2 type were shown in Table 4, accounted for 0.43% to 0.27% of fruiting body. And relative peak area of P1–P3 were shown in Table 4, accounted for 15.62% to 1.57% of fruiting body. Thus, there were little triterpenoids in GSP samples compared with fruiting body, which was matched up with the above results of HPTLC.

**Table 3:** LC–QTOF–MS data of P1-P3

Peaks	Types	[M−H]^−^ (*m/z*)	Formula	Error (ppm)	Collision energy (ev)	Fragments (*m/z*)
P1	d1	515.3004	C_30_H_43_O_7_^−^	2.1	35.5	515.2991 (55.3), 497.2890 (100), 479.2748 (6.02), 453.2995 (18.98), 435.2873 (11.96), 383.2590 (2.83), 301.1809 (26.32), 299.1657 (56.72), 285.1500 (61.11)
P2	d1′	513.2858	C_30_H_41_O_7_^−^	3	35.4	495.2731 (100), 451.2843 (79.31), 301.1812 (88.82), 283.1705 (40.88), 247.1344 (38.65)
P3	d2	515.299	C_30_H_41_O_7_^−^	4.7	35.5	497.2886 (100), 453.3001 (79.21), 303.1964 (75.93), 287.2004 (17.93), 285.1861 (29.13), 249.1501 (58.46)

**Table 4:** Relative peak area (%) of triterpenoids in representative samples by LC–QTOF–MS

Codes	P1	P2	P3	d1 or d1′	d2
GL	100.00	100.00	100.00	100.00	100.00
GSP-05	15.62	16.81	2.72	2.55	0.43
GSP-09	10.84	12.11	1.99	1.82	0.32
GSP-11	7.60	8.90	1.57	1.53	0.27

### HPTLC analysis of triterpenoids by bionic extraction

**Fig. 3:**
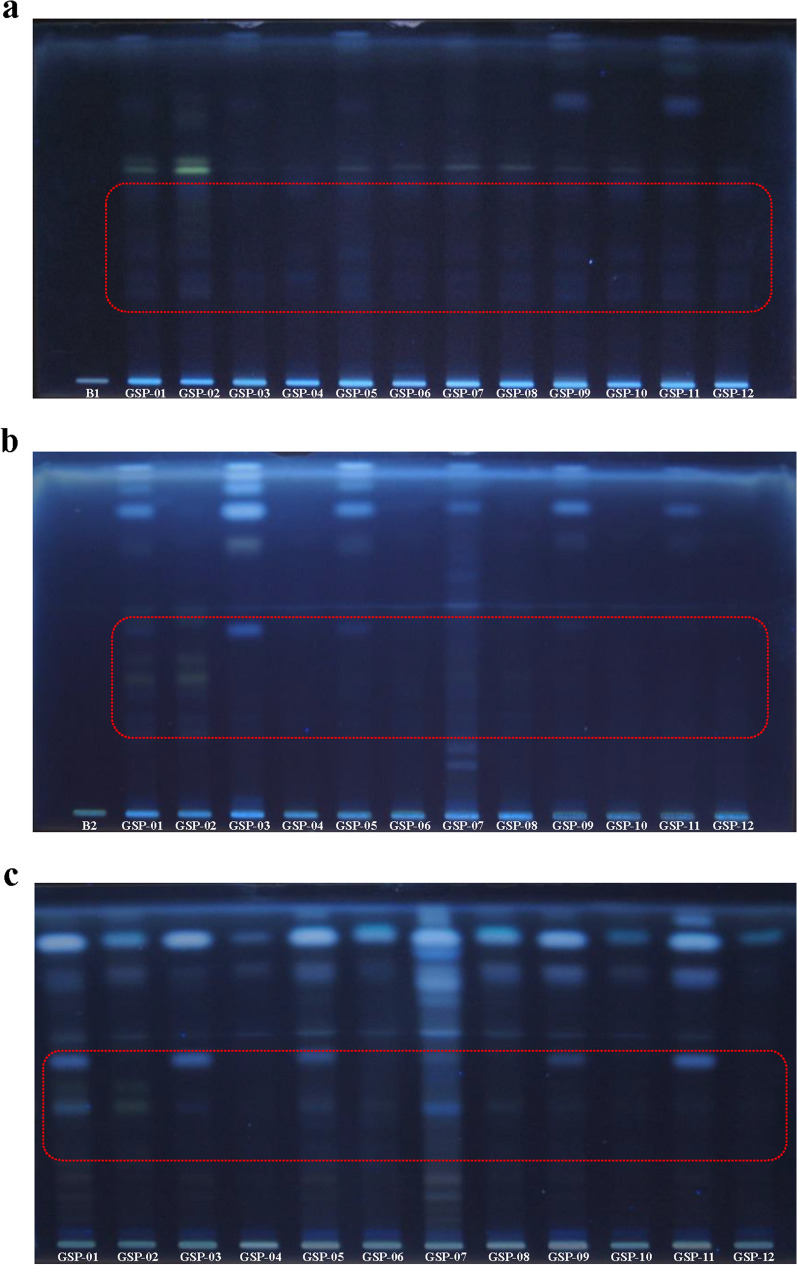
HPTLC chromatogram of GSP in artificial gastric fluid (**a**). HPTLC chromatogram of GSP in artificial intestinal fluid (**b**). HPTLC chromatogram of GSP after bionic extraction (**c**). B1—The blank of artificial gastric fluid; B2—The blank of artificial intestinal fluid

As demonstrated in Fig. 3a, b, respectively, there were no obvious band of triterpenoids in artificial gastric fluid, and only some faint bands at triterpenoids area in artificial intestinal fluid. The residues after bionic extraction were extracted in methanol again, as Fig. 3c shown, its dissolution of triterpenoids was higher than that in artificial gastrointestinal fluid. However, there were just a few bands at triterpenoids area, which were almost as same as Fig. 1a. Grayscale scanning integral was still used to profile the triterpenoids, and the relative peak area results demonstrated in Table 2. As Table 2 indicated, triterpenoids in GSP samples could not be dissolved in artificial gastrointestinal fluid, and most of triterpenoids were still in the residues.

## Discussion

HPTLC is a convenient and flexible method for routine qualitative analysis of traditional Chinese medicines with simple operation and clear results. Compared with HPLC–UV and colorimetric method, the two main methods of detecting triterpenoids in *Ganoderma* spore, HPTLC could simultaneously test multiple samples and avoid the interference of fatty acids in *Ganoderma* spore on colorimetric method. The fatty acids in the spore could react with perchloric acid, which would make the colorimetric result higher than the actual value. Meanwhile, it should be noticed that the results would also be higher if triterpenes were extra added into GSP samples. LC–QTOF–MS was used to further verified the triterpenoids and demonstrated that there were little triterpenoids in GSP samples, which was in accordance to the results of HPTLC. Therefore, it could be considered that GSP samples contained little triterpenes originally, and triterpenoids were not suitable to be quality control marker of GSP.

Bionic extraction is simulating human absorption and digestion process in artificial gastrointestinal fluid, which comprehensively reflects the principle of medical bionics and chemical bionics. The whole drug is integrated with bionic extraction and evaluated based on its markers to study traditional Chinese medicines, which elucidates the viewpoint of “entire effect” that traditional Chinese medicine theory always advocates. Thus, bionic extraction was applied on GSP to indicate the dissolution of triterpenoids in gastrointestinal tract. Triterpenoids in GSP samples could not be dissolved in artificial gastrointestinal fluid, and most of triterpenoids were still in the residues. It should have further study to improve the utilization of triterpenoids in human body. Besides, the types and contents of components in *Ganoderma* spore powder might change after bionic extraction. For example, compared with Fig. 1a, the peak area of a blue band (Rf = 0.56) was increased in each broken GSP residue of Fig. 3c. However, the result should be further investigated. Significantly, plenty of oily substance was found in broken GSP samples after extraction in methanol. Similarly, it had been demonstrated that the content of fat-soluble components such as triglycerides and fatty acids in GSP was much higher than that of triterpenoids [24]. And fatty acids in *Ganoderma* spore had anti-tumor effect [25, 26]. Therefore, the quality marker of GSP should be carefully considered, which could not totally follow that of *Ganoderma* fruiting body.

## Conclusions

In this study, GSP had been proven that it contained little triterpenoids. Therefore, triterpenoids should not be used as quality marker of GSP samples. This study was helpful to develop the quality control of GSP and its relative health-care products.

## Data Availability

The data in this study is available from the corresponding author upon reasonable request.

## Abbreviations

HPTLC: High-performance thin-layer chromatography
LC–QTOF–MS: Liquid chromatography coupled with electrospray ionization quadrupole-time-of-flight mass spectrometry
GL: *Ganoderma lucidum* Fruiting body
GSP: *Ganoderma* Spore powder
